# Folate Supplementation for Peripheral Neuropathy: A Systematic Review

**DOI:** 10.3390/nu17203299

**Published:** 2025-10-20

**Authors:** Ana Carolina Alves Maues, Mònica Gemma Moren Abat, María Benlloch, Gonzalo Mariscal

**Affiliations:** 1 OPKO Health Spain S.L.U., Palol de Revardit, 17843 Girona, Spain; 2 Department of Basic Biomedical Sciences, Catholic University of Valencia San Vicente Mártir, 46001 Valencia, Spain; 3 Institute for Research on Musculoskeletal Disorders, Catholic University of Valencia San Vicente Mártir, 46001 Valencia, Spain

**Keywords:** folate, folic acid, peripheral neuropathy, diabetic peripheral neuropathy

## Abstract

Background: Peripheral neuropathy (PN) represents a considerable and rapidly growing global health burden, with diabetic PN alone impacting nearly half of diabetic patients. Evidence from experimental studies highlighted that folate supplementation may protect nerve health by supporting myelin maintenance, minimizing oxidative stress, and enhancing neurotrophic factors. Nevertheless, its clinical efficacy and safety in managing PN have not yet been established. This study seeks to evaluate the role of folate in managing PN regarding the efficacy and safety endpoints. Methods: Up to July 2025, a comprehensive search of four electronic databases, encompassing PubMed, Scopus, Web of Science, and Cochrane Library, was executed, collecting studies evaluating the folate in managing PN. Outcomes included pain scores, symptom improvement endpoints, Neuropathy Total Symptom Score (NTSS) scores, epidermal nerve fiber density (ENFD), biomarkers, and side effects. Results: The narrative synthesis demonstrated consistent symptomatic benefits with pain reductions reaching 3 points, with symptom resolution rates of 87.5% and NTSS-6 score enhancements varying from 0.9 to 1.5 points. Notably, objective structural improvements in ENFD were observed, with increases reaching 97%. Furthermore, folate showed an excellent safety and tolerability profile with only one adverse event reported among 1367 individuals. Folate significantly decreased homocysteine and high-sensitivity C-reactive protein (hs-CRP) levels. Conclusions: Folate showed promising symptomatic benefits for peripheral neuropathy, with objective structural improvements (ENFD) and favourable biomarker changes (homocysteine, hs-CRP reduction), with an excellent safety profile.

## 1. Introduction

Peripheral neuropathy (PN) represents a significant and growing public health challenge, with an estimated prevalence of 2.4% in the general population, with estimates rising notably to about 8% among older adults. PN includes a wide range of clinical pathologies that mainly present with peripheral nervous system dysfunction [1]. Globally, diabetic peripheral neuropathy (DPN) is recognized as the most frequently encountered form of PN, affecting around 28% of adults in the United States [2,3]. Additionally, the longitudinal research has demonstrated that nearly 50% of patients with diabetes will develop DPN during their lifetime, with prevalence escalating with disease progression, rising from 26% five years post-diagnosis to 41% after ten years [4].

Patients with PN more commonly experience numbness and paresthesias, which are often accompanied by pain, muscle weakness, and reduced deep tendon reflexes. Typically, PN evolves over months to years, with some variants progressing more rapidly and severely. The pathophysiology of PN usually encompasses damage to the myelin-producing Schwann cells within peripheral nerves, especially in the segmental forms and to a lesser extent, in axonal types [5]. This disorder exhibits a wide range of severity and clinical features that reflect its involvement of motor, sensory, and autonomic fibers [6]. Notably, effective regeneration and continued maintenance of the myelin sheath after nerve injury are fundamental for the functional recovery of PN [5].

Peripheral neuropathy develops through a complex interplay of metabolic, vascular, and inflammatory mechanisms that result in axonal degeneration and demyelination. Chronic hyperglycemia, oxidative stress, and microvascular dysfunction contribute to Schwann cell injury, impaired axonal transport, and loss of protective myelin sheaths, leading to progressive sensory loss, pain, and motor deficits [5,6]. Despite this multifactorial pathogenesis, current standard treatments for PN remain largely symptom-focused. Pharmacologic options include serotonin–norepinephrine reuptake inhibitors (e.g., duloxetine), gabapentinoids (e.g., pregabalin), and tricyclic antidepressants, which provide variable pain relief but do not address underlying nerve damage or functional recovery [7,8]. Other agents, such as topical capsaicin and lidocaine patches, may reduce pain but are often limited by local irritation and short duration of effect. Importantly, there are no widely approved disease-modifying therapies that promote nerve regeneration or restore sensory function, underscoring the need to explore interventions with neuroprotective and regenerative potential, such as folate.

Folate deficiency has been identified as a potential risk factor for PN, with emerging evidence suggesting that even modestly low serum folate levels (6.8–13.5 nmol/L) may increase the risk of PN among adults [9,10]. Moreover, experimental studies have demonstrated a protective effect of folic acid against PN in diabetic rats by attenuating axonal degeneration and promoting remyelination [4]. Methyltetrahydrofolate reductase (MTHFR), an essential enzyme in folate metabolism, significantly influences plasma homocysteine and folate concentrations, and certain MTHFR gene polymorphisms have been linked to vascular complications in type 2 diabetes [11]. Beyond its metabolic role, folate exerts several neuroprotective effects that may be relevant for peripheral nerve health: it provides methyl groups necessary for DNA synthesis and repair, supports Schwann cell proliferation and myelin maintenance, and enhances neural stem cell differentiation and nerve growth factor (NGF) expression through activation of the mitogen-activated protein kinase (MAPK) pathway [12,13]. By lowering homocysteine, folate can reduce oxidative stress and endothelial dysfunction, improving microvascular perfusion and nitric oxide bioavailability, which are critical for nerve repair [14]. Deficiency of folic acid can impair nervous system development, limit neural stem cell function, and compromise nerve regeneration [15,16,17].

Among the various B-vitamins implicated in nerve health, folate has received particular attention because it is essential for one-carbon metabolism and methylation reactions critical to neuronal DNA repair and myelin synthesis, it lowers homocysteine (a neurotoxic and vasculopathic metabolite linked to nerve injury), and its biologically active form, L-methylfolate, readily crosses the blood–nerve barrier to support Schwann cell proliferation and axonal regeneration [4,9,10,11,12,13,14]. Observational and interventional studies also suggest that folate deficiency is independently associated with increased risk of peripheral neuropathy [9,10], making it a biologically and clinically compelling target.

While supplementation shows promise in enhancing neurodevelopment, reducing neuroinflammation, and promoting axonal repair, its clinical efficacy and safety in managing PN remain to be fully established. This study seeks to systemically synthesize the evidence from the available literature regarding the role of folate in managing PN.

## 2. Materials and Methods

This systematic investigation was executed with complete adherence to the guidelines declared in the Cochrane Handbook and PRISMA statement to guarantee an adequate execution and reporting of this systematic review and meta-analysis [18,19]. This study was prospectively registered under this identification number (https://doi.org/10.17605/OSF.IO/KFT2H, access date: 8 July 2025).

### 2.1. Criteria of Eligibility

Eligible studies included observational designs (prospective or retrospective) as well as randomized controlled trials, open-label, and single- or double-blind designs, written in English or Spanish. Acceptable comparators comprised placebo, no treatment, or non-drug interventions. Studies of all dosages, routes of administration, combinations, and durations were considered. Only human studies were included, involving male, female, or mixed populations. The study outcomes of interest were pain scores, symptomatic improvement outcomes, NTSS, its subdomains, epidermal nerve fiber density (ENFD), plasma biomarkers, and adverse events related to folate intervention. The exclusion criteria encompass conference abstracts, review articles, and foreign language studies.

### 2.2. Literature Search and Selection Process

The scope of our electronic search involved various databases, such as PubMed, Scopus, Web of Science, and Cochrane Library, from the origin of databases until July 2025. The detailed search strategy and results for each database are represented in Supplementary Table S1.

The gathered articles from the literature search were initially imported into EndNote v 21 (Clarivate Analytics, Philadelphia, PA, USA) to ensure proper identification and removal of duplicates. Consequently, two independent authors conducted a two-step screening process. First, the titles and abstracts of the eligible articles were evaluated using the Rayyan web [20]. Then, a comprehensive full-text assessment was employed for the relevant articles. Any emerging discrepancies among the authors were addressed through discussion.

### 2.3. Data Extraction

Two independent reviewers utilized a predesigned data extraction sheet to gather the relevant summary and baseline data, encompassing study design, country, recruitment period, folate active form and dose, sample size, age (years), male gender, body mass index (BMI), history of diabetic retinopathy, intervention period, outcomes, inclusion criteria, and conclusion. Discussion between the reviewers was employed to resolve any apparent conflicts.

### 2.4. Risk of Bias Assessment

The Cochrane Risk of Bias Two tool (ROB2, v2) was implemented to evaluate the risk of bias in RCTs [21]. The ROB2 tool comprehensively evaluates the quality of RCTs through critical methodological domains, including the randomization process, deviation from the planned interventions, incomplete outcome data, assessment of outcomes, selection of the reported results, and other sources of bias. Each RCT was categorized as having a low, some concerns, or high risk of bias, determined by a cumulative judgment score.

The Newcastle Ottawa scale (NOS) was utilized to evaluate the quality of double-arm observational studies [22]. It evaluated three essential domains, including population and exposure selection, comparability between cohorts, and outcomes assessment, with final evaluation for each study as having good, moderate, or poor quality. The National Institute of Health (NIH) tool was employed for assessing the quality of single-arm observational studies through the assessment of critical domains, such as a clear description of the study objective and population, exposure assessment more than once over time, and sufficiency of sample size [23]. Each study was finally classified as having good, fair, or poor quality.

### 2.5. Systematic Review Synthesis

Due to the marked variability among the included studies in terms of population characteristics, outcome measures, and follow-up durations, a meta-analytic approach was not appropriate. Instead, a narrative synthesis was conducted. For continuous variables, we summarized baseline, follow-up, and change data; for dichotomous variables, we reported improvement rates or changes in prevalence.

To address design-specific evidence evaluation, we stratified included studies by design type (randomized controlled trials vs. observational studies), presenting these stratifications in a Supplementary Table. This approach allows readers to assess the strength of evidence from RCTs separately from observational data.

To facilitate comparison across studies utilizing different measurement scales and to assess clinical relevance, we calculated standardized mean differences (SMD; Cohen’s d) for pain and neuropathic symptom outcomes where sufficient data were available. SMD effect sizes were interpreted as small (0.2), medium (0.5), or large (0.8) according to established conventions [24]. Additionally, we compared observed changes against minimal clinically important differences (MCID) derived from the literature: 2 points for pain scales (VAS/NRS 0-10) [25,26] and 1 point for NTSS-6 [27].

## 3. Results

### 3.1. Literature Search

**Figure 1:**
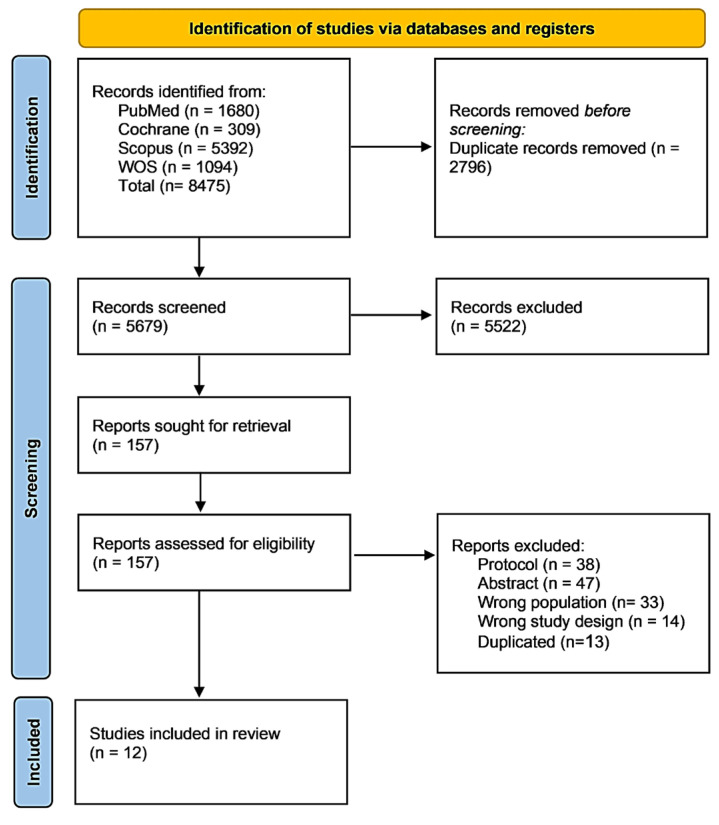
PRISMA (Preferred Reporting Items for Systematic Reviews and Meta-Analyses) flow diagram illustrating the systematic literature search and study selection process. The search was conducted across four electronic databases from database inception through July 2025, yielding a total of 8475 records. 12 studies met the inclusion criteria and were included in the final qualitative synthesis, comprising 3015 participants with peripheral neuropathy. Follow-up durations across included studies ranged from 2 to 12 months (median: 6 months).

A total of 8475 articles were retrieved from the search of four main databases; 2796 of them were eliminated as duplicates utilizing EndNote. As a result, 5679 articles were assessed through the title and abstract screening stage, excluding 5522 articles, and 157 articles proceeded to the full-text screening phase. Ultimately, 12 studies were included in this review, while 38 protocols, 47 conference abstracts, 33 studies with the wrong population, 14 studies with the wrong study designs, and 13 duplicates were excluded. Figure 1 illustrates the PRISMA flow diagram of the study selection process.

### 3.2. Characteristics of the Included Studies

This systematic review involved 3015 patients with PN, either diabetic or not, collected from three RCTs, eight prospective observational studies, and one retrospective observational study [28,29,30,31,32,33,34,35,36,37,38,39]. The included studies covered various geographical areas, with three studies conducted in Asia, two studies performed in Portugal, and seven studies conducted in the USA. L-methyl folate (with or without calcium) was the predominant form assessed, with a folate dose ranging from 0.4 to 15 mg/day. Folate was primarily administered in combination with other active substances. In several studies, folate was delivered as part of multicomponent formulations; for example, LMF-MC-PLP (containing L-methylfolate, methylcobalamin, and pyridoxal-5′-phosphate) and UMP + folic acid + B12. The mean age of our study population was 58.3 years [SD = 4], ranging from 54 to 65.5 years. In-depth summary and baseline information of the included studies are presented in Table 1.

### 3.3. Risk of Bias Results

**Figure 2:**
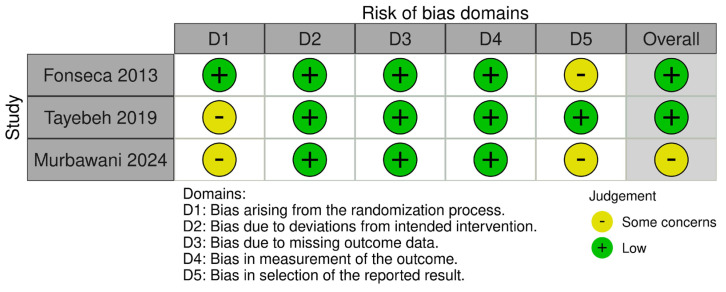
Risk of bias evaluation across the three included randomized controlled trials (RCTs) using the Cochrane Risk of Bias 2 (ROB2) tool. Color coding: green = low risk, yellow = unclear risk, red = high risk. Assessment domains include random sequence generation, allocation concealment, blinding, incomplete outcome data, selective reporting, and other sources of bias. The three RCTs included in this assessment evaluated folate-based interventions over follow-up periods of 4 to 6 months in patients with diabetic peripheral neuropathy (DPN), with primary outcomes including Neuropathy Total Symptom Score-6 (NTSS-6, scale 0–21), pain scores (Visual Analog Scale 0–10), and biomarker changes (homocysteine, inflammatory markers).

Three RCTs were assessed by the Cochrane ROB2 tool. Two studies were rated as having low risk of bias, and one showed some concerns due to issues related to the randomization process and selection of the reported outcomes, as outlined in Figure 2. Notably, NOS categorized two double-arm observational studies as having good quality, as shown in Supplementary Table S2. In the same context, NIH for single-arm observational studies rated four studies as having good quality and three as having fair quality, as presented in Supplementary Table S3.

### 3.4. Systematic Review

Design-stratified analysis (Supplementary Table S4) shows that RCT evidence (n = 6 treatment arms) demonstrates benefits with folate-based interventions, while a larger body of observational evidence (n = 11 studies) corroborates these findings. The heterogeneity in study populations, interventions, and outcome measures precluded meta-analysis but allowed for qualitative assessment of treatment effects across different neuropathy etiologies and folate formulations.

#### 3.4.1. Pain

Folate administration showed meaningful reductions in pain among patients with PN across studies (Table 2). For instance, Trippe et al. [36] reported a 1.8-point decrease in pain severity (32%) over the follow-up period. In a study by Negrao et al. [35], the pain intensity was reduced from 5.9 ± 2.0 to 3.9 ± 2.1 following folate administration, while 7 points decreased the Pain Disability Questionnaire (PDQ) total score throughout follow-up. Notably, Jacobs et al. [31] reported a 3-point pain reduction with folate intervention compared to just 0.25 in the controls. Nevertheless, Fonseca et al. [29] found a minimal visual analogue scale (VAS) improvement among patients prescribed folate (−0.27) relative to those administered a placebo (−0.03).

#### 3.4.2. Symptom Improvement

Folate-based interventions revealed promising results regarding symptom improvement, NTSS, and NTSS subdomains (Table 2 and Table 3). In a study by Jacob et al. [31], the symptom resolution rates reached 87.5% with folate intervention compared to 25% in controls. In contrast, McNamara et al. [32] reported a 30.9% % improvement in monofilament sensation, while Yukawa et al. [38] reported an improvement of 66.7% in the neurological symptoms. Considerable improvements in NTSS-6 scores were noticed across studies. For example, Trippe et al. [36] reported a 35% reduction (−1.5 points) in NTSS-6 scores with folate intervention. Conversely, Fonseca et al. [29] reported slightly lower reductions estimated at 0.9 and 0.96 points over 16 and 24 weeks, respectively.

The subdomain results of the NTSS demonstrated substantial reductions in common neuropathic symptoms. Trippe et al. [36] reported a 41.9% reduction in the prevalence of burning pain, a 42.9% reduction in deep aching pain, and a 35.9% reduction in lancinating pain. In Negrao et al. [35], the severe numbness decreased from 43.8% to 4.4% (90% reduction), and severe tingling fell from 35.4% to 6.3% (82% reduction). Additionally, the severe burning pain completely resolved (100% reduction) while the electric shock pain reduced by 88.1% [35].

Standardized mean difference analysis revealed predominantly medium to large effect sizes for both pain (SMD range: 0.5–1.5) and NTSS-6 outcomes (SMD range: 0.5–0.8), indicating clinically meaningful improvements across heterogeneous measurement scales (Supplementary Table S5). Comparison against established minimal clinically important differences demonstrated that 75% of studies reporting pain outcomes achieved reductions exceeding the MCID threshold of 2 points, while 67% of studies reporting NTSS-6 outcomes exceeded the MCID threshold of 1 point. These findings confirm that the observed treatment effects are not only statistically significant but also clinically relevant for patients with diabetic peripheral neuropathy.

#### 3.4.3. Epidermal Nerve Fiber Density

The current literature demonstrated modest enhancements in ENFD with folate-based intervention (Table 3. In McNamara et al. [32], the ENFD increases from 5.2 to 5.7 fibers/mm in the right foot (+ 11.5%) and from 4.7 to 5.7 in the left foot (+23.4%) following folate administration. Notably, the non-dominant limb showed a 20.8% increase in ENFD, while the dominant limb exhibited an 11.9% increase. Jacobs et al. [30] noticed a rise in the ENFD in the calf from 1.55 to 3.05 fibers/mm (97% increase), with 73% of patients showing increased ENFD.

#### 3.4.4. Biomarkers

Folate supplementation was consistently associated with favorable biomarker changes across the included clinical trials (Table 3). Fonseca et al. [29] reported marked increases in serum total folate (+7.25 nmol/L) and 5-methyltetrahydrofolate (5-MTHF) (+229.70 nmol/L) together with significant reductions in homocysteine (−2.7 μmol/L; *p* = 0.001) and methylmalonic acid (MMA) (−63.29 nmol/L), indicating improved one-carbon metabolism and reduced functional vitamin B12 deficiency. Tayebeh et al. [28] observed a modest but significant homocysteine reduction (−0.1 μmol/L; *p* < 0.05) accompanied by increased serum folate concentrations (+2 ng/mL; *p* < 0.05) and improvement in nerve conduction parameters, including increased peroneal and sural amplitudes and velocities with shortened latencies. Murbawani et al. [33] demonstrated an additional anti-inflammatory effect, showing a mean high-sensitivity C-reactive protein (hs-CRP) decrease of −1.8 mg/L compared with a +1.2 mg/L rise in the placebo group (*p* = 0.01). Importantly, none of the studies reported clinically significant vitamin B12 depletion, mitigating the concern of folate masking B12 deficiency. Beyond these trial data, emerging evidence highlights that folate may also modulate neuro regeneration and microvascular repair by enhancing nerve growth factor (NGF) and brain-derived neurotrophic factor (BDNF) expression, reducing oxidative stress markers such as malondialdehyde (MDA) and total antioxidant capacity (TAC), and improving endothelial nitric oxide bioavailability through tetrahydrobiopterin (BH4)-dependent nitric oxide synthase coupling [12,14,40]. Additionally, MTHFR C677T polymorphisms influence homocysteine response to folate supplementation and may predict treatment efficacy [41]. Collectively, these findings demonstrate that folate impacts both metabolic and neuro regenerative pathways, supporting its potential disease-modifying role in peripheral neuropathy.

#### 3.4.5. Adverse Events

Generally, the folate-based intervention was extremely safe and well tolerated across the included studies (Table 3). Only one adverse event was reported in 1367 participants (0.07%), with no serious events or withdrawals due to side effects.

## 4. Discussion

The present systematic review comprehensively synthesizes the available evidence on the clinical efficacy and safety of folate for managing PN, collecting the evidence from 12 studies comprising 3015 patients. The narrative synthesis demonstrated symptomatic benefits, with pain reductions up to 3 points on standardized scales and substantial declines in burning pain, deep aching pain, and electric-shock-like pain. Notably, the symptom resolution rates reached 87.5% in some studies, with 66.7% improvement in neurological symptoms in another study. NTSS reductions ranged from 0.9 to 1.5 points, representing clinically meaningful symptomatic changes. In contrast, objective structural improvements were observed in ENFD, with increases up to 97%. Furthermore, biomarkers synthesize indicated rises in folate levels and reductions in homocysteine and MMA. Generally, folate showed an excellent safety profile, with only one adverse event reported among 1367 participants.

The observed clinical improvements can be supported by several complementary biological actions of folate on peripheral nerves. Folate reduces homocysteine, a metabolite that promotes oxidative stress and endothelial dysfunction, thereby improving microvascular perfusion and nitric oxide bioavailability essential for nerve repair [14]. It also donates methyl groups required for DNA synthesis and repair, supporting Schwann cell proliferation and myelin sheath restoration [5,12]. In preclinical models, folate activates the MAPK pathway, increasing the expression of neurotrophic factors such as NGF and BDNF, which promote neuronal survival and axonal regeneration [12,40]. Moreover, folate mitigates inflammation and oxidative damage while enhancing neurogenesis [9,38]. Genetic factors, particularly the MTHFR C677T polymorphism, influence homocysteine metabolism and may modulate individual response to supplementation [41]. These mechanistic pathways suggest that folate acts beyond pain relief to support nerve repair and vascular health, reinforcing its potential as a disease-modifying therapy for peripheral neuropathy. These clinical results are biologically consistent with preclinical evidence. Animal models of diabetic neuropathy demonstrate that folate supplementation preserves small nerve fibers, enhances myelin thickness, and reduces oxidative and inflammatory damage [4,42]. Cellular experiments confirm that folate can directly stimulate Schwann cell growth and NGF secretion, accelerate axonal sprouting, and restore mitochondrial and endothelial function under hyperglycemic and oxidative conditions [12,13,14]. Such data reinforce the concept that folate acts beyond symptomatic pain control to support structural nerve repair.

While the current approved treatments for PN, such as duloxetine and pregabalin, offer considerable pain relief, they primarily target symptom management without addressing nerve regeneration or sensory recovery [7,8]. Therefore, there is a pressing need to investigate disease-modifying intervention that could enhance nerve regeneration and functional recovery in patients with PN. Folate emerged as a promising therapeutic candidate for PN by facilitating nerve regeneration and slowing disease progression rather than focusing on symptomatic relief only [12]. A randomized trial by Fonseca et al. [29] revealed that the combination therapy with L-methylfolate, methylcobalamin, and pyridoxal-5′-phosphate (LMF-MC-PLP) significantly improved the neuropathy symptoms in DPN patients, with NTSS-6 scores reduced by up to 0.96 points at 24 weeks (*p* = 0.033). Although the primary sensory endpoint (vibration perception threshold) was not met, the transient reduction in neuropathy-related disability (NSD reduction of −0.78 at 16 weeks) implies a functional benefit for folate-based combination beyond symptomatic relief. Mechanistically, the LMF-MC-PLP combination significantly reduced homocysteine by 2.68 µmol/L compared to an increase of 0.48 µmol/L in the placebo group (*p* = 0.001), potentially reducing the oxidative stress and restoring vascular function that provides a better vascular health for nerve regeneration [14]. Notably, the safety profile was excellent, with no significant adverse events reported related to the study intervention and no study withdrawals, indicating a high tolerability. Nevertheless, the trial’s short duration and the absence of considerable sensory changes emphasize a significant limitation demanding long-term data.

An Iranian experience involving 80 patients with DPN (40 in the folate group and 40 in the placebo group) provides promising results regarding the disease-modifying effects of folic acid supplementation on PN [28]. Over 16 weeks, the daily administration of 1 mg of folic acid significantly increased the serum folate concentrations (8.1 vs. 10.1; *p* < 0.001) and reduced homocysteine levels (2.2 vs. 2.1; *p* < 0.001), supporting a plausible mechanism involving the suppression of oxidative stress pathways. Notably, these biochemical improvements translated to objective enhancements in nerve conduction parameters. In the folate group, sensory sural amplitude improved notably (3.3 vs. 2.4; *p* < 0.001) relative to placebo group, along with substantial gains in motor nerve function, encompassing increased amplitudes (e.g., peroneal 1.5 vs. 0.9; *p* = 0.001), conduction velocities (peroneal 30.2 vs. 29.9; *p* = 0.002), and decreased onset latencies (peroneal 5.4 vs. 5.2; *p* = 0.019). Although not restoring the nerve conduction velocity to normal values, these significant observations rigorously suggest that folic acid may modify the underlying disease process, not just provide symptomatic relief.

It is worth stating that beyond homocysteine-lowering, folate offers considerable neuroprotection effects through mitigating inflammation and oxidative damage and by promoting the expression of the nerve growth factor, which is crucial for neuronal regeneration and survival [4,40,42]. This trial also found no notable impact of folate on serum vitamin B12, emphasizing a reassuring safety profile that counters the prevalent concern of folate masking B12 deficiency [43].

Another observational experience by McNamara et al. [32] investigating the effects of the compound LMF-MC-PP within 123 patients with DPN strongly supported the available evidence regarding the potential disease-modifying effects of folate. Over 6 months of intervention, ENFD improved significantly, with a mean gain varying from 0.6 to 1.1 fibers/mm, contrasting notably with the expected annual decline of −0.68 fibers/mm/year reported in untreated DPN cohorts [32]. These structural enhancements were concomitant with meaningful clinical gains in sensation. For instance, the monofilament testing exhibited a substantial improvement, with 60 patients (48.8%) with intact sensation at baseline increasing to 95 (77.2%) after intervention, and of 63 patients with absent sensation at baseline, 38 (60.3%) restored intact sensation by 6 months. This robust parallel enhancement in ENFD and monofilament sensation highlights the likely disease-modifying effect of folate-based intervention rather than merely symptomatic improvement. Nevertheless, the study’s observational nature and the absence of a control group restrict the ability to draw definitive conclusions. Notably, using objective and quantifiable markers like ENFD strengthens confidence in the structural effects of folate.

Furthermore, the analgesic effects of folate therapy were also evident across several studies [29,31,35]. In a study by Negrao and his colleagues [35], The combination of a nutritional supplement containing uridine monophosphate, folic acid, and vitamin B12 significantly improved pain outcomes and had an excellent safety profile in patients with peripheral entrapment neuropathy. Patients experienced a significant reduction in global pain scores, from 17.3 ± 5.9 at baseline to 10.3 ± 6.1 at final assessment after two months, suggesting a considerable effect for this combination in reducing the burden of neuropathic pain. Notably, this supplement decreased the severity of neuropathic symptoms, as severe tingling or pricking declined from 35.4% of patients at baseline to just 6.3% at final assessment, while severe numbness fell from 43.8% to 4.4%. These significant improvements could be attributed to the synergistic action of nucleotides, which promotes the peripheral myelin sheath repair, and the roles of folic acid and vitamin B12 in accelerating nerve regeneration and the repair process [34,44,45].

### 4.1. Strengths and Drawbacks

This systematic review investigation provides the most extensive and updated evidence regarding the role of folate in managing PN, gathering the evidence from 12 studies incorporating 3015 patients. A key strength of this study is the inclusion of studies that were conducted across several geographical areas and distinct populations, supporting the generalizability and credibility of the findings. However, like any present research, this study is not free of limitations. First, the significant heterogeneity of population heterogeneity, outcomes assessment, and follow-up durations across the included studies hindered us from performing a meta-analysis. Second, including observational studies may have introduced selection bias and residual confounding, tempering our conclusions’ certainty.

A notable strength of our analysis is the assessment of clinical significance through standardized mean differences and comparison with established minimal clinically important difference thresholds. Despite the inability to perform quantitative meta-analysis due to methodological heterogeneity, the consistency of effect sizes and the high proportion of studies exceeding MCID thresholds provide robust evidence for clinically meaningful treatment effects. This approach addresses the challenge of interpreting results across studies employing different measurement scales and reinforces the clinical relevance of our findings beyond statistical significance alone.

Our design-stratified synthesis reveals important methodological considerations. While RCT evidence provides the highest certainty regarding treatment effects, the consistency observed across different study designs strengthens confidence in the therapeutic potential of folate-based interventions. However, the heterogeneity in interventions (different folate formulations, doses, and combination products) and outcome measures highlights the need for standardized approaches in future research. The diversity in study populations suggests potential applicability across various neuropathy etiologies, though dedicated trials in specific populations would strengthen the evidence base.

### 4.2. Advances in Clinical Knowledge and Future Suggestions

This systematic review expands clinical knowledge by demonstrating that folate-based interventions provide both symptomatic benefits and objective structural improvements in PN. Specifically, objective parameters such as nerve conduction velocity and epidermal nerve fiber density showed measurable improvements, suggesting potential disease-modifying effects beyond the symptomatic benefits observed in pain and neuropathic symptom scores. The excellent safety and tolerability profiles further support its applicability in clinical settings.

Nevertheless, the observed limitations across the included studies, such as significant heterogeneity, shorter follow-up durations, and reliance on observational studies, emphasize the demand for future large-scale, placebo-controlled randomized studies. These trials should focus on examining long-term functional and structural outcomes and investigating the optimal dosing strategies and combinations for better management of PN.

## 5. Conclusions

Folate-based regimens show a promising signal of symptomatic benefit in peripheral neuropathy, with several studies also suggesting objective improvements in nerve structure or function (ENFD, nerve conduction). The most consistent evidence comes from combination formulations (L-methylfolate with vitamins B12/B6 or UMP), while monotherapy data remains limited but biologically supportive. Randomized trials confirm homocysteine reduction and NTSS-6 improvement, though heterogeneity and small sample sizes temper certainty. Across 1367 participants reporting safety, only one mild adverse event was documented, suggesting an excellent safety profile.

## Figures and Tables

**Table 1:** Showing summary and baseline characteristics of included studies and population:.

Study ID	Study Design	Country	Recruitment Period	Folate Form	Folate Dose (mg/Day)	Group	Intervention	Sample Size	Age, Mean (SD) (Years)	Male Sex, *n* (%)	BMI, Mean (SD) kg/m^2^	History of Diabetic Retinopathy, *n* (%)	Treatment Period (Month)	Outcomes	Inclusion Criteria	Conclusion
Fonseca 2013 [29]	Double-blinded RCT	USA	between June 2008 and May 2010	L-methyl folate calcium	6	Folate	Metanx (a combination of LMF-MC-PLP)	106	62.29 (8.54)	73 (68.90)	NA	17 (16)	6	NTSS-6, plasma levels of folate, vitamin B12, homocysteine, and High-sensitivity C-reactive protein.	Patients aged 25 to 80 years with type 2 DM and neuropathy (baseline VPT: 25–45 volts at hallux on either leg)	In patients with diabetes, LMF-MC-PLP (Metanx) significantly improves DPN symptoms and quality of life measurements with low adverse events
						Control	Placebo	108	62.95 (9.17)	75 (69.40)	NA	19 (17.6)				
Jacobs 2011 [30]	Prospective observational	USA	between July 2008 and March 2009	L-methyl folate	6	Folate	A combination of LMF-MC-PLP	11	60.45 (13.10)	4 (36.36)	NA	NA	6	ENFD	Patients with Type 2 DM with a history of both positive and negative sensory symptoms of the lower extremities	Administration of LMF-MC-PLP promotes an increase in ENFD in participants with diabetic small fiber neuropathy and improves symptoms toms of anesthesia, paresthesia, or dysesthesia
Jacobs 2013 [31]	Prospective observational	USA	NA	L-methyl folate	6	Folate	Metanx (a combination of LMF-MC-PLP) + pregabalin therapy	16	NA	7 (43.75)	NA	NA	5	Numeric rating scale	Diabetic patients who experienced a partial response (individual receiving pregabalin for longer than 4 months, with only partial resolution of burning paresthesia of the feet) after pregabalin therapy for DPN	Patients with partial symptom resolution after pregabalin showed significant pain relief after the addition of LMF-MC-PLP (Metanx)
						Control	pregabalin therapy alone	8		3 (37.50)	NA					
McNamara 2016 [32]	Prospective observational	USA	between 2010 and 2014	L-methyl folate calcium	6	Folate	Metanx (a combination of LMF-MC-PLP)	123	NA	74 (60.20)	NA	NA	6	ENFD and monofilament testing	Patients with type 2 diabetes and diagnosed with DPN based on vibratory sensorium, warm–cold discrimination, or loss of protective sensorium	LMF-MC-PLP (Metanx) significantly improves ENFD and monofilament testing after 6 months in patients with DPN
Murbawani 2024 [33]	RCT	Indonesia	between September 2018 and March 2019	Folic acid	1	Folate	combination of 1 mg folic acid, 400 ug vitamin B12, and 10 mg vitamin B6	36	NA	11 (30.60)	NA	NA	4	High-sensitivity C-reactive protein	45–65 years old diabetic neuropathy patients	Supplementation of folic acid, vitamin B6, and vitamin B12 combination is able to significantly decrease the inflammatory status in diabetic neuropathy patients
						Control	Placebo	39		8 (20.50)						
Negrão 2014 [34]	Prospective observational	Portugal	between 2 January 2012 and 30 April 2012	Folic acid	0.4	Folate	Keltican (UMP [50 mg], folic acid [400 μg], and vitamin B12 [3 μg]	212	59 (14.4)	81 (38.20)	27.30	NA	2	Pain DETECT questionnaire	Patients diagnosed with painful peripheral neuropathy, irrespective of the cause of the disease	In patients with neuropathic pain associated with peripheral neuropathy, supplementation with the combination of UMP, vitamin B12, and folic acid was effective in reducing neuropathic pain
Negrão 2016 [35]	Prospective observational	Portugal	NA	Folic acid	0.4	Folate	Keltican (UMP [50 mg], folic acid [400 μg], and vitamin B12 [3 μg]	48	56 (12.40)	11 (22.90)	26.9 (4.10)	NA	2	Pain DETECT questionnaire	Patients diagnosed with clinical symptoms of painful peripheral entrapment neuropathy, irrespective of the cause	UMP + vitamin B12 + folic acid administered to patients with peripheral entrapment neuropathy resulted in a significant reduction in pain
Tayebeh 2019 [28]	Double-blinded RCT	Iran	between June 2017 and April 2018.	Folic acid	1	Folate	1 mg folic acid	40	54.90 (5.50)	25 (62.50)	26 (4.80)	10 (25)	4	Serum folic acid, Vitamin B12, and homocysteine, and electromyography nerve conduction studies	Patients with confirmed diabetic neuropathy by electromyography-nerve conduction studies	Folic acid supplementation decreased serum levels of homocysteine and increased serum levels of folic acid
						Control	Placebo	40	55.30 (6)	18 (45)	25 (5.10)	14 (35)				
Trippe 2016 [36]	Prospective observational	USA	between November 2010 and April 2012	L-methyl folate	NA	Folate	Metanx (a combination of LMF-MC-PLP)	544	65.50 (10.90)	252 (46.30)	NA	NA	3	NTSS-6	Patients with diabetic peripheral neuropathy, at least 18 years old, and naive to treatment with LMF-MC-PLP.	Patients with diabetic peripheral neuropathy treated with LMF-MC-PLP (Metanx) achieved significant improvements in total symptom score (NTSS-6) and in quality of life and functioning, together with greater medication satisfaction.
Wade 2012 [37]	Retrospective observational	USA	between 1 January 2004 and 31 July 2010	L-methyl folate	NA	Folate	Metanx (a combination of LMF-MC-PLP)	814	54 (7.01)	460 (56.51)	NA	92 (11.30)	12	hospitalization risk (all-cause and disease-related) and direct healthcare costs (all cause and disease-related)	Patients aged 18 to 64 years with 2 or more medical claims for type 2 diabetes or 1 pharmacy claim for antidiabetic agents, and with at least 1 medical claim for peripheral neuropathy	LMF-MC-PLP (Metanx) use among patients with DPN was associated with lower hospitalization risk and lower disease-related costs.
						Control	Control	814	55 (7.16)	441 (54.18)	NA	84 (10.32)				
Walker 2010 [39]	Prospective observational	USA	between June 2006 and October 2007	L-methyl folate	6	Folate	Metanx (a combination of LMF-MC-PLP)	20	NA	NA	NA	NA	12	PSSD	Patients with type 2 diabetes with complaints consistent with DPN	In patients with DPN, LMF-MC-PLP (Metanx) could restore cutaneous sensitivity
Yukawa 2001 [38]	Prospective observational	Japan	NA	Folate (not specified)	15	Folate	Folic acid (15 mg/d)	36	55.5 (19.30)	NA	NA	NA	2	serum folic acid	Patients with neurological disease who have folate deficiency	Neurological symptoms were more frequently improved by folate supplementation in patients with neuropathy than without it

Abbreviations: RCT: Randomized controlled trial, LMF-MC-PLP: L-methyl folate, methyl cobalamin, and pyridoxal-5′-phosphate, NTSS-6: Neuropathy Total Symptom Score, DPN: Diabetic peripheral neuropathy, ENFD: epidermal nerve fiber density, PSSD: Pressure-Specified Sensory Device; NA: Not applicable; UMP: uridine monophosphate.

**Table 2:** Peripheral Neuropathy Treatment Outcomes Summary.

VAS/NRS/Pain Scores.					
Study	Number of Patients	Scale Used	Baseline Mean ± SD	Follow-Up Mean ± SD	Change from Baseline
Trippe 2016 [36]	544	Pain Severity (0–10)	5.80	4	−1.80 (32% reduction)
Negrao 2016 [35]	48	PDQ Total Score	17.30 ± 5.90	10.30 ± 6.10	−7.00 ± 6.00
Negrao 2016 [35]	48	Pain Intensity (Visit)	5.90 ± 2.00	3.90 ± 2.10	−2.00 ± 2.10
Negrao 2014 [34]	212	PDQ Total Score	17.50 ± 5.70	8.80 ± 5.20	−8.7 ± 5.5
Negrao 2014 [34]	212	Pain Intensity (Visit)	6.60 ± 2.00	3.70 ± 1.80	−2.9 ± 1.9
Jacobs 2013 [31]	16	Pain Score (0–10)	3.94	0.94	−3
Jacobs 2013 [31]	8	Pain Score (Control)	3.63	3.38	−0.25
Fonseca 2013 [29]	106	VAS (Treatment)	3.26 ± 2.77	-	−0.27 ± 2.28
Fonseca 2013 [29]	108	VAS (Placebo)	3.25 ± 2.76	-	−0.03 ± 2.61
Symptom Improvement					
Study	Number of Patients	Outcome Measured	Improved/Total	Improvement Rate (%)	Control Rate (%)
McNamara 2016 [32]	123	Monofilament Sensation Improvement	38/123	30.90%	10% (historical)
Yukawa 2001 [38]	36	Neurological Symptoms	24/36	66.70%	10% (historical)
Jacobs 2011 [30]	11	Paresthesias/Dysesthesias	9/11	82.00%	10% (historical)
Jacobs 2013 [31]	16	Symptom Resolution (Treatment)	14/16	87.50%	-
Jacobs 2013 [31]	8	Symptom Resolution (Control)	2/8	25.00%	-
NTTS Scores					
Study	Number of Patients	Scale	Baseline Mean ± SD	Follow-up Time	Change from Baseline
Trippe 2016 [36]	544	NTSS-6	4.30 ± 1.50	12 weeks	−1.50 ± 1.80 (35% reduction)
Fonseca 2013 [29] (16 w)	106	NTSS-6 (Treatment)	3.73 ± 1.79	16 weeks	−0.90 ± 1.42
Fonseca 2013 [29] (16 w)	108	NTSS-6 (Placebo)	3.45 ± 2.05	16 weeks	−0.40 ± 1.72
Fonseca 2013 [29] (24 w)	106	NTSS-6 (Treatment)	3.73 ± 1.79	24 weeks	−0.96 ± 1.54
Fonseca 2013 [29] (24 w)	108	NTSS-6 (Placebo)	3.45 ± 2.05	24 weeks	−0.53 ± 1.69
NTTS Subdomains (Individual Symptoms)					
Study	N	Symptom	Baseline Prevalence (%)	Follow-up Prevalence (%)	Reduction (%)
Trippe 2016 [36]	544	Numbness	67%	47%	29.80%
Trippe 2016 [36]	544	Prickling/Tingling	89%	61%	31.50%
Trippe 2016 [36]	544	Burning Pain	81%	47%	41.90%
Trippe 2016 [36]	544	Deep Aching Pain	70%	40%	42.90%
Trippe 2016 [36]	544	Lancinating Pain	64%	41%	35.90%
Trippe 2016 [36]	544	Allodynia	63%	47%	25.40%
Negrao 2016 [35]	48	Severe Numbness	43.80%	4.40%	90.00%
Negrao 2016 [35]	48	Severe Tingling	35.40%	6.30%	82.20%
Negrao 2016 [35]	48	Electric Shock Pain	36.10%	4.30%	88.10%
Negrao 2016 [35]	48	Severe Burning	18.80%	0%	100%

**Table 3:** Epidermal Nerve Fiber Density, Biomarkers, and Safety Outcomes.

ENFD (Epidermal Nerve Fiber Density)						
Study	Number of Patients	Location	Baseline (Fibers/mm)	Follow-Up (Fibers/mm)	Change (Fibers/mm)	Change (%)
McNamara 2016 [32]	122	Right Foot	5.20 ± 5.20	5.70 ± 6.00	+0.60 ± 3.70	11.50%
McNamara 2016 [32]	122	Left Foot	4.70 ± 4.40	5.70 ± 5.80	+1.10 ± 3.40	23.40%
McNamara 2016 [32]	111	Dominant Limb	5.10 ± 5.10	5.70 ± 5.90	+0.60 ± 3.80	11.80%
McNamara 2016 [32]	110	Non-dominant Limb	4.80 ± 4.40	5.90 ± 5.80	+1.00 ± 3.40	20.80%
McNamara 2016 [32]	117	Pooled (4 locations)	4.95 ± 4.80	5.75 ± 5.90	+0.83 ± 3.60	16.80%
Jacobs 2011 [30]	11	Calf	1.55 ± 1.98	3.05 ± 2.68	+1.50 ± 1.50	97%
Jacobs 2011 [30]	11	Patients with ENFD Increase	-	-	8/11 patients	73%
Biomarkers						
Study	Number of Patients	Biomarker	Baseline (Treatment)	Baseline (Control)	Change (Treatment)	Change (Control)
Fonseca 2013 [29]	214	Homocysteine (μmol/L)	9.71 ± 4.29	9.47 ± 3.90	−2.70 ± 2.90	+0.58 ± 2.58
Fonseca 2013 [29]	214	Total Folate (nmol/L)	42.19 ± 9.93	43.04 ± 9.30	+7.25 ± 10.52	−1.07 ± 8.27
Fonseca 2013 [29]	214	5-MTHF (nmol/L)	59.68 ± 31.32	60.73 ± 31.35	+229.70 ± 163.42	−2.13 ± 25.15
Fonseca 2013 [29]	214	MMA (nmol/L)	186.16 ± 120.11	195.72 ± 169.19	−63.29 ± 107.00	−15.42 ± 59.90
Tayebeh 2019 [28]	80	Homocysteine (μmol/L)	2.20 ± 0.20	2.20 ± 0.10	−0.1 (0.2) Significant reduction	0 (0.173) No change
Tayebeh 2019 [28]	80	Folate (ng/mL)	8.10 ± 1.10	7.80 ± 1.70	+2.00 ± 1.30	−0.10 ± 1.80
Murbawani 2024 [33]	75	Hs-CRP (mg/L)	6.30 ± 6.09	4.90 ± 4.54	−1.80 ± 4.80	+1.20 ± 5.70
Adverse Events						
Study	Number of Patients	Treatment Duration	Adverse Events (n)	Serious AE (n)	Withdrawals due to AE (n)	AE Rate (%)
McNamara 2016 [32]	123	6 months	0	0	0	0.00%
Tayebeh 2019 [28]	80	16 weeks	0	0	0	0.00%
Trippe 2016 [36]	544	12 weeks	0	0	0	0.00%
Yukawa 2001 [38]	36	60 days	0	0	0	0.00%
Negrao 2016 [35]	48	60 days	0	0	0	0.00%
Negrao 2014 [34]	212	60 days	0	0	0	0.00%
Jacobs 2011 [30]	11	6 months	0	0	0	0.00%
Fonseca 2013 [29]	214	24 weeks	1	1	0	0.47%
Jacobs 2013 [31]	24	20 weeks	0	0	0	0.00%
Murbawani 2024 [33]	75	4 weeks	0	0	0	0.00%
Total	1367	Variable	1	1	0	0.07%

## Data Availability

Not applicable.

## Supplementary Materials

The following supporting information can be downloaded at: https://www.mdpi.com/article/10.3390/nu17203299/s1, Supplementary Table S1: Detailed Search Results; Supplementary Table S2: Newcastle Ottawa Scale for observational studies; Supplementary Table S3: NIH for single-arm studies; Supplementary Table S4: Comprehensive Stratified Evidence Summary; Supplementary Table S5. Standardized Mean Differences, Minimal Clinically Important Differences, and Responder Rates for Pain and Neuropathic Symptom Outcomes.
