# Supplementary material for: Folate Supplementation for Peripheral Neuropathy: A Systematic Review
Source: Nutrients. 2025 Oct 20;17(20):3299. doi: 10.3390/nu17203299 (PMC12566604; doi:10.3390/nu17203299)
Supplement: Supplementary file 1 [file nutrients-17-03299-s001.zip › nutrients-3919700-supplementary-Tables S4 and S5.pdf]

Supplementary Table S4: Comprehensive Stratified Evidence Summary

Study ID	Study Design	Population/Diagnosis	Intervention	N	Duration (months)	Key Outcomes	Main Findings	Conclusion
**A. RANDOMIZED CONTROLLED TRIALS (n=6 treatment arms)**								
**Fonseca 2013 [30]**	Double-blinded RCT	Patients aged 25 to 80 years with type 2 DM and neuropathy (baseline VPT: 25-45 volts at hallux on e	Metanx (a combination of LMF-MC-PLP)	106	6	NTSS-6, plasma levels of folate, vitamin B12, homocystein e, and High-sensitivity C-reactive protein.	In patients with diabetes LMF-MC-PLP (Metanx) significantly improve DPN symptoms and quality of life measurements with l	In patients with diabetes LMF-MC-PLP (Metanx) significantly improve DPN symptoms and quality of life measurements with low adverse events
**Fonseca 2013 [30]**	Double-blinded RCT	Patients aged 25 to 80 years with type 2 DM and neuropathy (baseline VPT: 25-45 volts at hallux on e	Placebo	108	6	NTSS-6, plasma levels of folate, vitamin B12, homocystein e, and High-sensitivity C-reactive protein.	In patients with diabetes LMF-MC-PLP (Metanx) significantly improve DPN symptoms and quality of life measurements with l	In patients with diabetes LMF-MC-PLP (Metanx) significantly improve DPN symptoms and quality of life measurements with low adverse events
**Murbawani 2024 [34]**	RCT	45-65 years old diabetic neuropathy patients	combination of 1 mg folic acid, 400 ug vitamin B12, and 10 mg vitamin B6	36	4	High-sensitivity C-reactive protein	supplementation of folic acid, vitamin B6, vitamin B12 combination is able to significantly decrease inflammatory status	supplementation of folic acid, vitamin B6, vitamin B12 combination is able to significantly decrease inflammatory status in

								diabetic neuropathy patien
**Murbawani 2024 [34]**	RCT	45-65 years old diabetic neuropathy patients	Placebo	39	4	High-sensitivity C-reactive protein	supplementation of folic acid, vitamin B6, vitamin B12 combination is able to significantly decrease inflammatory status	supplementation of folic acid, vitamin B6, vitamin B12 combination is able to significantly decrease inflammatory status in diabetic neuropathy patien
**Tayebeh 2019 [29]**	Double-blinded RCT	patients with confirmed diabetic neuropathy by electromyography-nerve conduction studies	1 mg folic acid	40	4	Serum folic acid, Vitamin B12 and homocysteine, and electromyography nerve conduction studies	folic acid supplementation decreased serum levels of homocysteine and increased serum levels of folic acid	folic acid supplementation decreased serum levels of homocysteine and increased serum levels of folic acid
**Tayebeh 2019 [29]**	Double-blinded RCT	patients with confirmed diabetic neuropathy by electromyography-nerve conduction studies	Placebo	40	4	Serum folic acid, Vitamin B12 and homocysteine, and electromyography nerve conduction studies	folic acid supplementation decreased serum levels of homocysteine and increased serum levels of folic acid	folic acid supplementation decreased serum levels of homocysteine and increased serum levels of folic acid
**B. OBSERVATIONAL STUDIES - PROSPECTIVE (n=9 studies)**								
**Jacobs 2011 [31]**	Prospective observational	Patients with Type 2 DM with a history of both positive and negative sensory symptoms of the lower e	A combination of LMF-MC-PLP	11	6	ENFD	Administration of LMF-MC-PLP promotes increase in ENFD in participants	Administration of LMF-MC-PLP promotes increase in ENFD in participants

							with diabetic small fiber neuropathy and improves	with diabetic small fiber neuropathy and improves symp toms of anesthesia, pare
**Jacobs 2013 [32]**	Prospective observational	Diabetic patients who experienced partial response (individual receiving pregabalin for longer than	Metanx (a combination of LMF-MC-PLP) + pregabalin therapy	16	5	Numeric rating scale	Patients with partial symptoms resolution after pregabalin showed significant pain relief after addition of LMF-MC-PLP (	Patients with partial symptoms resolution after pregabalin showed significant pain relief after addition of LMF-MC-PLP (Metanx)
**Jacobs 2013 [32]**	Prospective observational	Diabetic patients who experienced partial response (individual receiving pregabalin for longer than	pregabalin therapy alone	8	5	Numeric rating scale	Patients with partial symptoms resolution after pregabalin showed significant pain relief after addition of LMF-MC-PLP (	Patients with partial symptoms resolution after pregabalin showed significant pain relief after addition of LMF-MC-PLP (Metanx)
**McNamara 2016 [33]**	Prospective observational	Patients with type 2 diabetes and diagnosed with DPN based on vibratory sensorium, warm-cold discrim	Metanx (a combination of LMF-MC-PLP)	123	6	ENFD, and monofilament testing	LMF-MC-PLP (Metanx) significantly improve ENFD and monofilament testing after 6 months in patients	LMF-MC-PLP (Metanx) significantly improve ENFD and monofilament testing after 6 months in patients

							with DPN	with DPN
**Negrão 2014 [35]**	Prospective observational	patients diagnosed with painful peripheral neuropathy, irrespective of the cause of the disease	Keltican (UMP [50 mg], folic acid [400 µg] and vitamin B12[3 µg]	212	2	Pain DETECT questionnaire	In patients with neuropathic pain associated with peripheral neuropathy, supplementation with the combination of UMP, vi	In patients with neuropathic pain associated with peripheral neuropathy, supplementation with the combination of UMP, vitamin B12, and folic acid was
**Negrão 2016 [36]**	Prospective observational	patients diagnosed with clinical symptoms of painful peripheral entrapment neuropathy, irrespective	Keltican (UMP [50 mg], folic acid [400 µg] and vitamin B12[3 µg]	48	2	Pain DETECT questionnaire	UMP + vitamin B12 + folic acid administered to patients with peripheral entrapment neuropathy resulted in significantly	UMP + vitamin B12 + folic acid administered to patients with peripheral entrapment neuropathy resulted in significantly reduction of pain
**Trippe 2016 [37]**	Prospective observational	patients with diabetic peripheral neuropathy at least 18 years old and naive to treatment with LMF-M	Metanx (a combination of LMF-MC-PLP)	544	3	NTSS-6	patients with diabetic peripheral neuropathy treated with LMF-MC-PLP (Metanx) achieved significant improvements in total	patients with diabetic peripheral neuropathy treated with LMF-MC-PLP (Metanx) achieved significant improvements in total symptom score (NTSS-6) and in

**Walker 2010 [40]**	Prospective observational	patients with type 2 diabetes with complaints consistent with DPN	Metanx (a combination of LMF-MC-PLP)	20	12	PSSD	In patients with DPN LMF-MC-PLP (Metanx), could restore cutaneous sensitivity	In patients with DPN LMF-MC-PLP (Metanx), could restore cutaneous sensitivity
**Yukawa 2001 [39]**	Prospective observational	Patients with neurological disease who have folate deficiency	Folic acid (15mg/d)	36	2	serum folic acid	Neurological symptoms were more frequently improved by folate supplement in patients with neuropathy than without it	Neurological symptoms were more frequently improved by folate supplement in patients with neuropathy than without it
**C. OBSERVATIONAL STUDIES - RETROSPECTIVE (n=2 studies)**								
**Wade 2012 [38]**	Retrospective observational	Patients aged 18 to 64 years with 2 or more medical claims for type 2 diabetes or 1 pharmacy claim f	Metanx (a combination of LMF-MC-PLP)	814	12	hospitalization risk (all cause and disease related) and direct healthcare costs (all cause and dis	LMF-MC-PLP (Metanx) use among patients with DPN was associated with lower hospitalization risk and lower disease-related	LMF-MC-PLP (Metanx) use among patients with DPN was associated with lower hospitalization risk and lower disease-related costs.
**Wade 2012 [38]**	Retrospective observational	Patients aged 18 to 64 years with 2 or more medical claims for type 2 diabetes or 1 pharmacy claim f	Control	814	12	hospitalization risk (all cause and disease related) and direct healthcare costs (all cause and dis	LMF-MC-PLP (Metanx) use among patients with DPN was associated with lower hospitalization risk and	LMF-MC-PLP (Metanx) use among patients with DPN was associated with lower hospitalization risk and

			lower disease-related	lower disease-related costs.

RCT = randomized controlled trial; DPN = diabetic peripheral neuropathy; LMF = L-methylfolate; MC = methylcobalamin; PLP = pyridoxal-5-phosphate; VAS = Visual Analog Scale; NRS = Numeric Rating Scale; PSSD = Pressure-Specified Sensory Device; NIS-LL = Neuropathy Impairment Score-Lower Limbs; NTSS-6 = Neuropathy Total Symptom Score-6.

Supplementary Table S5. Standardized Mean Differences, Minimal Clinically Important Differences, and Responder Rates for Pain and Neuropathic Symptom Outcomes

A. Pain Outcomes

Study	N	Scale	Baseline Mean ± SD	Change from Baseline Mean ± SD	SMD (95% CI)	Exceeds MCID (≥2 points)	Responder Rate (%)
**Trippe 2016 [37]**	544	Pain Severity (0-10)	5.8	-1.8	Not calculable	No	NR
**Negrao 2016 [36]**	48	Pain Intensity (0-10)	5.9 ± 2.0	-2.0 ± 2.1	Large	Yes	NR
**Negrao 2016 [36]**	48	PDQ Total Score	17.3 ± 5.9	-7.0 ± 6.0	Large	Yes	NR
**Negrao 2014 [35]**	212	Pain Intensity (0-10)	6.6 ± 2.0	-2.9 ± 1.9	Large	Yes	NR
**Negrao 2014 [35]**	212	PDQ Total Score	17.5 ± 5.7	-8.7 ± 5.5	Large	Yes	NR
**Jacobs 2013 (Treatment) [32]**	16	Pain Score (0-10)	3.9	-3.0	Not calculable	Yes	NR
**Jacobs 2013 (Control) [32]**	8	Pain Score (0-10)	3.6	-0.2	Not calculable	No	NR
**Fonseca 2013 (Treatment) [30]**	106	VAS (0-10)	3.3 ± 2.8	-0.3 ± 2.3	Trivial	No	NR
**Fonseca 2013 (Placebo) [30]**	108	VAS (0-10)	3.2 ± 2.8	-0.0 ± 2.6	Trivial	No	NR

B. NTSS-6 (Neuropathic Total Symptom Score) Outcomes

Study	N	Scale	Baseline Mean ± SD	Change from Baseline Mean ± SD	SMD (95% CI)	Exceeds MCID (≥1 point)	Responder Rate (%)
**Trippe 2016 [37]**	544	NTSS-6 (0-21)	4.3 ± 1.5	-1.5 ± 1.8	Large	Yes	NR
**Fonseca 2013 (Treatment) 16w [30]**	106	NTSS-6 (0-21)	3.7 ± 1.8	-0.9 ± 1.4	Medium	No	NR
**Fonseca 2013 (Placebo) 16w [30]**	108	NTSS-6 (0-21)	3.5 ± 2.0	-0.4 ± 1.7	Small	No	NR
**Fonseca 2013**	106	NTSS-6 (0-	3.7 ± 1.8	-1.0 ± 1.5	Medium	No	NR

**(Treatment) 24w** [30]		21)					
**Fonseca 2013 (Placebo) 24w** [30]	108	NTSS-6 (0-21)	3.5 ± 2.0	-0.5 ± 1.7	Small	No	NR
