# Supplementary material for: Folate Supplementation for Peripheral Neuropathy: A Systematic Review
Source: Nutrients. 2025 Oct 20;17(20):3299. doi: 10.3390/nu17203299 (PMC12566604; doi:10.3390/nu17203299)
Supplement: Supplementary file 1 [file nutrients-17-03299-s001.zip › Supplementary Table 1.pdf]

**Supplementary Table S1: Detailed Search Results:**

| Database | Search term | Search field | Search results |
|---|---|---|---|
| PubMed | (Metanx OR folate OR folic OR methylfolate) AND (neuropath* OR pain) | All fields | 1,680 |
| Scopus | (Metanx OR folate OR folic OR methylfolate) AND (neuropath* OR pain) | Article title, Abstract, Keywords | 5,392 |
| WOS | (Metanx OR folate OR folic OR methylfolate) AND (neuropath* OR pain) | All fields | 1,094 |
| Cochrane Central | (Metanx OR folate OR folic OR methylfolate) AND (neuropath* OR pain) | All Text | 309 |

Search date: 30/5/2025
