# Supplementary material for: Folate Supplementation for Peripheral Neuropathy: A Systematic Review
Source: Nutrients. 2025 Oct 20;17(20):3299. doi: 10.3390/nu17203299 (PMC12566604; doi:10.3390/nu17203299)
Supplement: Supplementary file 1 [file nutrients-17-03299-s001.zip › Supplementary Table 2.pdf]

Supplementary Table S2: Newcastle Ottawa Scale for observational studies:

Study ID	Selection				Comparability	Outcome			Quality Score
	Representativeness of the exposed cohort	Selection of the non-exposed cohort	Ascertainment of exposure	Demonstration that the outcome of interest was not present at the start of the study	Comparability of cohorts on the basis of the design or analysis	Assessment of outcome	Was follow-up long enough for outcomes to occur	Adequacy of follow up of cohorts	
Jacobs 2013	*	*	*	*	*	*	*	*	Good quality
Wade 2012	*	*	*	*	**	*	*	*	Good quality
