# Supplementary material for: Folate Supplementation for Peripheral Neuropathy: A Systematic Review
Source: Nutrients. 2025 Oct 20;17(20):3299. doi: 10.3390/nu17203299 (PMC12566604; doi:10.3390/nu17203299)
Supplement: Supplementary file 1 [file nutrients-17-03299-s001.zip › Supplementary Table 3.pdf]

Supplementary Table S3: NIH for single-arm studies:

Study ID	1. Was the study question or objective clearly stated?	2. Was the study population clearly specified and defined?	3. Was the participation rate of eligible people at least 50%?	4. Were all the subjects selected or recruited from the same or similar populations (including the same time period)? Were inclusion and exclusion criteria for being in the study prespecified and applied uniformly to all participants?	5. Was sample size justification, power description, or variance and effect estimates provided?	6. For the analyses in this paper, were the exposure(s) of interest measured prior to the outcome(s) being measured?	7. Was the timeframe sufficient so that one could reasonably expect to see an association between exposure and outcome if it existed?	8. For exposures that can vary in amount or level, did the study examine different levels of the exposure as related to the outcome (e.g., categories of exposure, or exposure measured as continuous variable)?	9. Were the exposure measures (independent variables) clearly defined, valid, reliable, and implemented consistently across all study participants?	10. Was the exposure(s) assessed more than once over time?	11. Were the outcome measures (dependent variables) clearly defined, valid, reliable, and implemented consistently across all study participants?	12. Were the outcome assessors blinded to the exposure status of participants?	13. Was loss to follow-up after baseline 20% or less?	14. Were key potential confounding variables measured and adjusted statistically for their impact on the relationship between exposure(s) and outcome(s)?	Quality Rating (Good, Fair, or Poor)
Jacobs 2011	Yes	Yes	Yes	Yes	NR	NA	Yes	NA	Yes	Yes	Yes	NR	Yes	No	Good quality
McNamara 2016	Yes	Yes	Yes	Yes	NR	NA	Yes	NA	Yes	Yes	Yes	NR	Yes	No	Good quality
Negrão 2014	Yes	Yes	Yes	Yes	No	NA	No	NA	Yes	Yes	Yes	NR	Yes	No	Fair quality
Negrão 2016	Yes	Yes	Yes	Yes	No	NA	No	NA	Yes	Yes	Yes	NR	Yes	No	Fair quality
Trippe 2016	Yes	Yes	Yes	Yes	NR	NA	Yes	NA	Yes	Yes	Yes	NR	Yes	No	Good quality
Walker 2010	Yes	Yes	Yes	Yes	NR	NA	Yes	NA	Yes	Yes	Yes	NR	Yes	No	Good quality
Yikawa 2001	Yes	Yes	Yes	Yes	NR	NA	No	NA	Yes	No	Yes	NR	Yes	Yes	Fair quality
